# Supplementary material for: Association between oral microbiome and seven types of cancers in East Asian population: a two-sample Mendelian randomization analysis
Source: Front Mol Biosci. 2023 Nov 21;10:1327893. doi: 10.3389/fmolb.2023.1327893 (PMC10702768; doi:10.3389/fmolb.2023.1327893)
Supplement: Supplementary file 1 [file DataSheet1.ZIP › Supplementary Materials/MR plots for tongue/tongue═╝/Gastric cancer/pheno.1644_to_gastric cancer_forest.pdf]

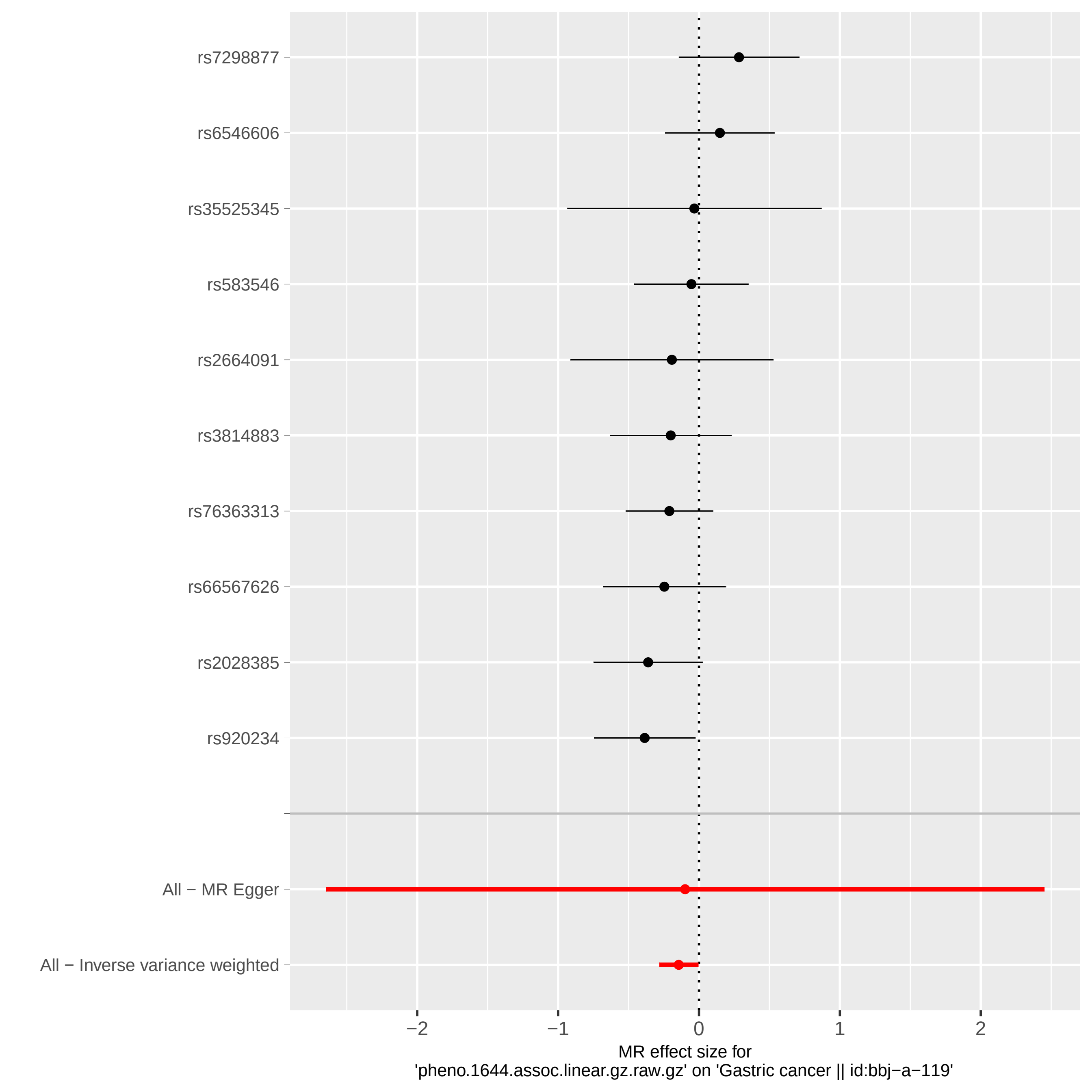

rs7298877
rs6546606
rs35525345
rs583546
rs2664091
rs3814883
rs76363313
rs66567626
rs2028385
rs920234
All − MR Egger
All − Inverse variance weighted
−2
−1
0
1
2
MR effect size for
'pheno.1644.assoc.linear.gz.raw.gz' on 'Gastric cancer || id:bbj−a−119'
